# Laboratory-acquired Buffalopox Virus Infection, India

**DOI:** 10.3201/eid2002.130358

**Published:** 2014-02

**Authors:** Thachamvally Riyesh, Shanmugasundaram Karuppusamy, Bidhan C. Bera, Sanjay Barua, Nitin Virmani, Sarita Yadav, Rajesh K. Vaid, Taruna Anand, Manish Bansal, Praveen Malik, Inderjeet Pahuja, Raj K. Singh

**Affiliations:** Veterinary Culture Collection, Hisar, India (T. Riyesh, S. Karuppusamy, B.C. Bera, S. Barua, R.K. Vaid, T. Anand, M. Bansal, P. Malik);; National Research Centre on Equines, Hisar (N. Virmani, I. Pahuja, R.K. Singh);; Central Institute for Research of Buffaloes, Hisar (S. Yadav)

**Keywords:** buffalopox virus, viruses, vaccinia virus, biosafety, laboratory infection, laboratory accident, researcher, India

**To the Editor:** In India, buffalopox virus (BPXV), a variant of vaccinia virus, is associated with severe disease outbreaks among buffaloes (*1**,**2*), cattle (*3*), and humans in contact with these animals (*1**,**4*). Most human BPXV infections occur in animal attendants and milkers (*1**,**4*). A similar type of vaccinia virus infection has also been reported from rural areas in Brazil (*5*). We report a case of laboratory-acquired infection with BPXV in a researcher in India. Clinical signs, symptoms, diagnosis, and management of this case highlight the need for observance and enforcement of strict biosafety measures within the laboratory.

A 28-year-old man (researcher) who was freeze-drying BPXV isolates in a laboratory in Hisar, India sustained a cut on his right palm through nitrile gloves by accidental piercing of shrapnel from a broken ampule. The virus being freeze-dried (10^5.5^ 50% tissue culture infectious doses/mL) was isolated from a buffalo in Jalgaon, India, in 2010. The injured site on the palm was immediately cleaned with 70% ethanol and treated with povidone–iodine solution. No untoward reaction was observed ≤2 days postinjury. Erythema appeared at the injury site on postinjury day 3. Subsequently, a small vesicle developed on postinjury day 5 (Figure, panel A). This vesicle progressed into a pustule with a central area of necrosis by postinjury day 7 (Figure, panel B). On postinjury day 9, symptoms worsened (onset of high fever and general malaise and pain at the affected site), and the researcher sought medical care.

**Figure:**
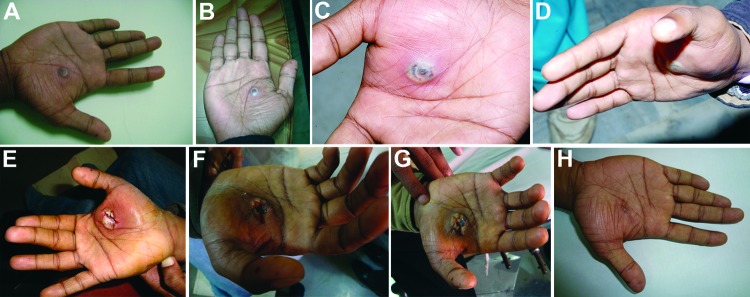
Progression of lesion on palm of researcher caused by buffalopox virus infection, India. A) Small vesicle on postinjury day 5. B) Pustule with a central area of necrosis on postinjury day 7. C) Pustule with edema on postinjury day 9. D) Increase in edema on postinjury day 10 (cyanosis is not apparent). E) Lesion after surgical excision on postinjury day 11. F) Lesion on postinjury day 19 (healing was erratic). G) Blackening and thickening around surgical site on postinjury day 30, which then extended to a wider circumference. H) Healed lesion with a black eschar on postinjury day 85.

Physical examination showed high fever (104°F), unilateral axillary lymphadenopathy, and edema of the palm (Figure, panel C). Amoxicillin (500 mg, 2×/d), cephalexin (500 mg, 2×/d), and analgesic/antipyretic (paracetamol, 500 mg, 2×/d) were prescribed to control secondary complications caused by bacterial infection and pain.

The next day, the entire palm became cyanotic and edema increased (Figure, panel D). The researcher was then referred to a specialty hospital where the lesion was surgically excised on postinjury day 11 (Figure, panel E) under axial block anesthesia. Blood, necrotic tissue, pustular material, and swab specimens were obtained for laboratory examination. Postsurgery treatment included cleaning of the surgical site on alternate days and oral medication (amoxicillin/clavulanic acid, 625 mg; ibuprofen, 400 mg; paracetamol, 325 mg; and rabeprazole, 20 mg) for 5 days.

On postinjury day 19, the surgeon advised the patient to take cefuroxime (500 mg/d for 5 days) and use a topical ointment containing mupirocin to prevent a delay in healing (Figure, panel F). The lesion healed slowly, and by postinjury day 30, thickening and blackening of the skin was observed (Figure, panel G) that extended to a wider area by postinjury day 38, and the skin started to peel off by postinjury day 50. The entire skin of the palm sloughed off with complete healing by postinjury day 85, leaving a 20-mm blackened eschar over the area (Figure, panel H).

Clinical samples were subjected to laboratory examination. Virus was isolated from tissue samples in a Vero cell line during the first passage. BPXV infection was confirmed by PCR amplification of orthopoxvirus-specific A type inclusion gene (552 bp) and a BPXV-specific C18L gene (368 bp) from tissue material and the laboratory-isolated virus (BPXV/Human/Lab/11), according to procedures described by Singh et al. (*6*). Sequences of these 2 genes were submitted to GenBank under accession nos. JN653284.1 and JN653278.1, respectively. Phylogenetic analysis showed 95%–100% nt similarity of the laboratory isolate (BPXV/Human/Lab/11) with other BPXVs from India (Technical Appendix Figure). Antibodies against BPXV were detected in patient serum samples by using an indirect immunoperoxidase test and a 50% plaque-reduction neutralization test according to methods reported by Bera et al. (*7*). Serum of the infected patient showed 50% plaque-reduction neutralization test titers of 256 and 512 on postinjury days 11 and 28, respectively. These findings confirmed BPXV infection because the patient had not been vaccinated against smallpox.

Freeze-drying of the glass ampule to −80°C caused a hairline crack in the glass. The ampule broke while being introduced into the freeze-drying manifold and pierced the palm of the researcher. As a follow-up measure, the freeze drying procedure was reviewed and the pre-freezing temperature was reduced to −60°C. Measures were also taken to ensure use of better-quality ampules. Surgical and contact material associated with the lesion was placed in biohazard bags for autoclaving before disposal. In addition, laboratory and hospital staff was apprised of the risk associated with BPXV transmission.

Reporting of laboratory-acquired infections is crucial because infections could also spread to other personnel. Strict biosafety practices and laboratory guidelines are useful in minimizing laboratory-acquired infections. Guidelines, no matter how stringent, are not sufficient on their own. Laboratory-acquired infections occur because humans or machines are not infallible. Thus, laboratories should have emergency procedures in place to deal with such situations.

Technical Appendix Phylogenetic trees of A-type inclusion and C18L genes buffalopox virus and related viruses.
