# Supplementary material for: Laboratory-acquired Buffalopox Virus Infection, India
Source: Emerg Infect Dis. 2014 Feb;20(2):325–7. doi: 10.3201/eid2002.130358 (PMC3901488; doi:10.3201/eid2002.130358)
Supplement: Technical Appendix — Phylogenetic trees of A-type inclusion and C18L genes buffalopox virus and related viruses. [file 13-0358-Techapp-s1.pdf]

Article DOI: http://dx.doi.org/10.3201/eid2002.130358

Laboratory-acquired Buffalopox Virus Infection, India

Technical Appendix

Phylogenetic Trees of A-Type Inclusion Genes and C18L Genes of Buffalopox Virus and Related Viruses

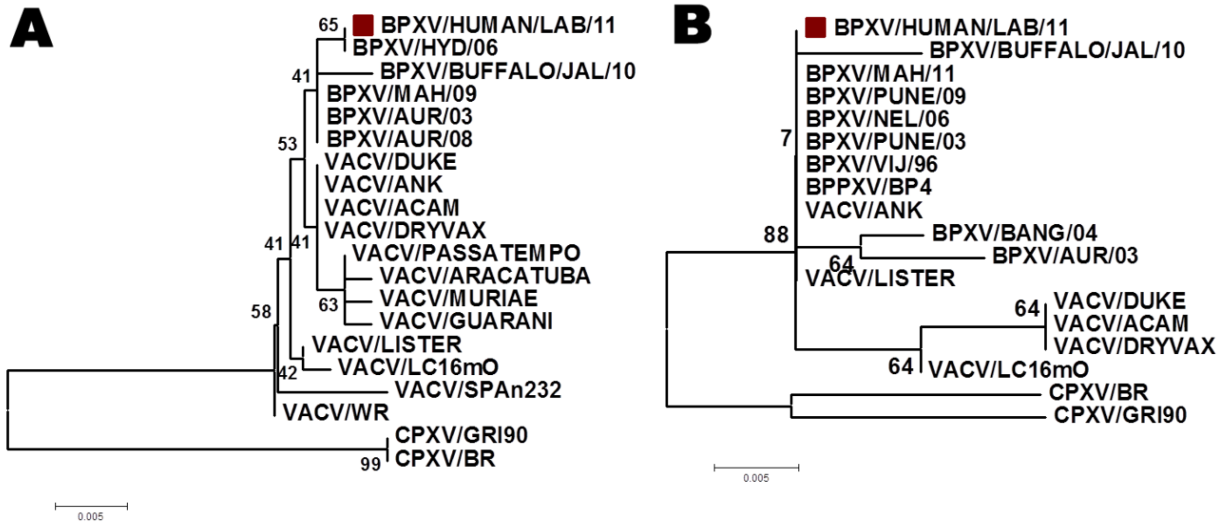

Technical Appendix Figure. Phylogenetic trees of A type inclusion genes (A) and C18L genes (B) of buffalopox virus and related viruses. Trees were constructed by using MEGA5 software (http://megasoftware.net/). Boxes indicate strain isolated in this study. Values along branches indicate bootstrap confidence calculated per 1,000 bootstrap values. Scale bars indicate nucleotide substitutions per site. BPXV, buffalopox virus; LAB/11, laboratory human isolate in 2011; HYD/06, buffalo isolate from Hyderabad in 2006; JAL/10, buffalo isolate from Jalgaon in 2010; MAH/09, human isolate from Maharashtra in 2009; AUR/03 and AUR/08, buffalo isolates from Aurangabad in 2003 and 2008, respectively; VACV, vaccinia virus; DUKE, Duke strain; ANK, Ankara strain; ACAM, vaccinia virus strain Acambis; DRYVAX, Dryvax strain; PASSATEMPO, vaccinia-like virus strain Passatempo from Brazil; ARACATUBA, vaccinia-like virus strain Aracatuba from Brazil; MURIAE, vaccinia virus strain from Brazil; GUARANI, vaccinia virus strain from Brazil; Lister, vaccinia virus strain; LC16mO, vaccinia virus strain; SPAn232, vaccinia virus strain from Brazil; WR, vaccinia virus strain Western Reserve; CPXV, cowpox virus; GRI90, cowpox virus strain; BR, Brington Red; MAH/11, human isolate from Maharashtra in 2011; PUNE/03 and PUNE/09, buffalo isolates from Pune in 2003 and 2009, respectively; NEL, buffalo isolate from Nellore in 2006; VIJ/96, buffalo isolate from Vijaywada in 1996; BP4, reference strain of buffalopox virus; BANG/04, buffalo isolate from Bangladesh in 2004.
